# Population Pharmacokinetic Study of Cefazolin Used Prophylactically in Canine Surgery for Susceptibility Testing Breakpoint Determination

**DOI:** 10.3389/fphar.2018.01137

**Published:** 2018-10-09

**Authors:** Petra Cagnardi, Federica Di Cesare, Pierre-Louis Toutain, Alain Bousquet-Mélou, Giuliano Ravasio, Roberto Villa

**Affiliations:** ^1^Department of Health, Animal Science and Food Safety, Università degli Studi di Milano, Milan, Italy; ^2^The Royal Veterinary College, University of London, London, United Kingdom; ^3^INTHERES, Université de Toulouse, INRA, ENVT, Toulouse, France; ^4^Department of Veterinary Medicine, Università degli Studi di Milano, Milan, Italy

**Keywords:** cefazolin, dog, prophylactic administration, surgery, population pharmacokinetics, PK/PD cut off value

## Abstract

This study aimed to determine the population pharmacokinetic (Pop PK) parameters of cefazolin administered prophylactically at 25 mg/kg intravenously (IV) 30 min before surgery in a canine population of 78 dogs and assess whether covariates, such as sex, age, body weight (BW), breed, health status, creatinine level, and surgery time, have an influence on cefazolin disposition. The ultimate goal was to compute PK/PD cut off values and subsequently establish a specific clinical breakpoint (CBP) for the development of an antimicrobial susceptibility test (AST) of cefazolin in dogs according to the VetCAST approach. Two to 11 blood samples were collected from each dog from 5 to 480 min after cefazolin administration. A two-compartment model was selected, and parameterization was in terms of serum clearance (CL), intercompartmental CL(s) (Q) and volume(s) of distribution (V). The percentage of cefazolin binding to serum protein was 36.2 ± 5.3%. Population primary parameter estimates V1, V2, CL, and Q were (typical value ± SE) 0.116 ± 0.013 L/kg, 0.177 ± 0.011 L/kg, 0.0037 ± 0.0002 L/kg/min, and 0.0103 ± 0.0013 L/kg/min, respectively. Cefazolin presented rapid distribution and elimination half-lives (mean ± SE) 4.17 ± 0.77 min and 57.93 ± 3.11 min, respectively. The overall between-subject variability (BSV) for estimated primary parameters ranged from 36 to 42%, and none of the seven explored covariates were able to reduce this variability by an amplitude clinically relevant. By Monte Carlo simulation, the probability of a PK/PD target attainment (here to achieve a free serum concentration exceeding the MIC for 50% of the dosing interval in 90% of dogs) was computed with a dosage of 25 mg/kg administered IV every 6 h for 4 administrations in 24 h. The computed PK/PD cut off value was 2 mg/L. In conclusion, cefazolin administered prophylactically in surgical dogs at 25 mg/kg IV every 6 h was deemed effective against pathogens with a MIC value ≤ 2 mg/L and from a PK/PD perspective, can be recommended in a wide range of canine patient populations with no necessary dose adjustment for special dog subpopulations.

## Introduction

In animals as well as in human medicine, surgical site infection (SSI) represents a dangerous complication that can easily lead to an extension of hospital stay and an increase in medical costs (Whittem et al., 1999; Hauser et al., 2006; Xu et al., 2015). The perioperative administration of antimicrobial drugs (AMDs) can decrease the incidence of SSIs (Brown et al., 1997; Prospero et al., 2011; Xu et al., 2015); however, to avoid serious consequences, such as the risk of hospital-acquired infection and the selection of antimicrobial-resistant bacterial strains, administration has to be carried out carefully and appropriately (Song and Glenny, 1998; Knights et al., 2012). In veterinary surgery, depending on the surgery site, pathogens, such as *Staphylococcus* spp, *Streptococcus* spp, *Enterobacteriaceae* and *Pasteurella*, are commonly encountered (Boothe and Boothe, 2015). Cefazolin is a first-generation cephalosporin with good activity against gram-positive cocci (*Staphylococcus* spp, including beta-lactamase-producing strains, and *Streptococcus* spp), many *Enterobacteriaceae* (*E. coli, Klebsiella* spp., *Proteus mirabilis*), *Pasteurella* spp and anaerobes (Papich and Riviere, 2009). Thus, cefazolin has become one of the most commonly AMD used perioperatively, and it has been recommended as the ideal prophylactic AMD for dogs undergoing surgery based on its spectrum, low toxicity and cost (Rosin et al., 1993; Marcellin-Little et al., 1996; Gonzalez et al., 2017). Cephalosporins like all beta-lactams have time-dependent killing activity; therefore, antibiotic plasma concentrations should be kept above the minimum inhibitory concentration (MIC) as long as possible during therapy. This index is defined as T > MIC, and it has been established that a T > MIC of 40–50% of the dosing interval is adequate for a successful outcome. More precisely, as only unbound drug concentration is available for antimicrobial activity, the index should be measured as the duration of free plasma concentration exceeding the MIC and is reported as *f*T > MIC (Turnidge, 1998; Toutain et al., 2002; Papich, 2014). Recently for cefazolin, a breakpoint of efficacy against bacteria isolated from animals was set at ≤ 2 mg/L (Papich, 2014; Clinical Laboratory Standards Institute [CLSI], 2015). Cefazolin pharmacokinetics (PK) has been extensively studied in dogs with a classical approach (Richardson et al., 1992; Rosin et al., 1993; Petersen and Rosin, 1995; Marcellin-Little et al., 1996; Singh et al., 1998; Harika et al., 2001; Gonzalez et al., 2017). Following this, it has been recommended that time-dependent AMD should be readministered every 2 half-lives during surgery to maintain targeted plasma concentrations (Marcellin-Little et al., 1996; Plumb, 2011; Gonzalez et al., 2017).

Population pharmacokinetics (Pop PK) is widely applied to define the sources of PK variability in target patient populations, thus identifying and assessing demographic, pathophysiological, environmental, and drug-related factors that can influence drug disposition (Ette and Williams, 2004; Kiang et al., 2012). Population modeling is also used in clinical trials, where the participants are representative of the real treated population, in contrast to healthy subjects or highly selected patients in traditional PK studies (Riviere, 2009; Bon et al., 2017). Moreover, through a Pop PK approach with a sufficient knowledge of covariates, it is possible to predict a typical PK profile for any given patient, allowing the definition of the correctness of the treatment (Concordet et al., 2004). In veterinary medicine, over the last decades, many Pop PK studies regarding AMDs have been performed in dogs (Regnier et al., 2003; Zhao et al., 2012; Prados et al., 2014; Hnot et al., 2015; Papich, 2017), but no study has been carried out with cefazolin. Starting from these assumptions, the aim of the study was to determine the Pop PK profile of cefazolin administered prophylactically at 25 mg/kg by intravenous (IV) bolus 30 min before surgery in a representative canine population to identify whether covariates such as sex, age, body weight (BW), breed, health status, creatinine level and surgery time, have an influence on cefazolin disposition. Furthermore, the ultimate goal was to compute PK/PD cut off values to establish a specific clinical breakpoint (CBP) for the development of an antimicrobial susceptibility test (AST) of cefazolin in dogs, according to the VetCAST approach (Toutain et al., 2017).

## Materials and Methods

### Animals

With ethical approval (Organismo Preposto al Benessere Animale, OPBA_23_2016) and after obtaining the owners’ written consent, 78 client-owned dogs were enrolled for the study. Dogs were of different breeds, sexes, ages, BWs and healthy or presenting concomitant diseases, all scheduled at the University Veterinary Hospital of Milan for any type of surgical procedure. In all subjects, type of surgery, duration of the procedure, anesthetic and analgesic perioperative protocol and medical history were recorded. For each dog, the blood count and biochemical profile were evaluated.

### Sample Collection and Analysis

Before drugs administration, 1 mL of venous blood was collected through an angio-venous catheter (Surflo^®^, 20 G, 32 mm IV catheter Terumo, Vetefarma Srl, Cuneo, Italy) previously placed into a peripheral vein. For surgery, each dog was premedicated, generally with sedatives (i.e., α2-agonists) and opioids (i.e., methadone) and put under general anesthesia, induced by propofol and maintained with isoflurane in 100% oxygen. Thirty min after the surgical procedures, meloxicam (0.2 mg/kg, Metacam, Boehringer Italia, Milan, Italy) was administered subcutaneously as an analgesic drug. Cefazolin (Cefazolina Teva, Teva Italia S.r.l., Milan, Italy) was administered via IV bolus to all dogs at 25 mg/kg 30 min before surgery. For cefazolin quantification, venous blood samples (1 mL each) were collected from each subject at prefixed times (5, 10, 15, 30, 45, 60, 90, 120, 150, 180, 240, 360, and 480 min). Then, all samples were centrifuged (4000 *g*, 10 min) to obtain serum and frozen at -20°C pending analysis.

Cefazolin was extracted and quantified by HPLC-UV from canine serum samples according to the published method by Kunicki and Was (2012) with slight modifications (see **Supplementary File S1** for details). The analytical method was validated in our hands in compliance with the recommendations defined by the European Community (European Commission, 2002; Commission decision 2002/657/EC) and with the international guidelines (European Medicine Agency [EMA], 2011 – VICH GL49). The calibration curves were prepared with 6 spiked solutions obtained diluting the original stock solution of cefazolin (1 mg/mL) in canine blank serum to achieve concentrations ranging from 0.2 to 20 μg/mL. The correlation coefficients (r) resulted > 0.99 for 2 replicates. The precision (repeatability) and accuracy were determined by analyzing blank samples (*n* = 6 for each concentration) that were spiked with 0.2, 2, or 20 μg/mL of cefazolin. The results fell within the accepted ranges for precision (6.75, 13.9, and 13.5% for 0.2 μg/mL, 2 μg/mL, and 20 μg/mL, respectively) and accuracy (2.5, -3.42, and 2.66% for 0.2 μg/mL, 2 μg/mL and 20 μg/mL, respectively). A LOQ value of 0.2 μg/mL was set. The LOD was 0.00024 μg/mL. The specificity of the method was demonstrated by the absence of interference in 20 blank serum samples at the cefazolin retention time. In the case of samples above the upper limit of quantification (20 μg/mL), these samples were quantified upon dilution.

The percentage of cefazolin serum protein binding was determined *in vitro* with ultrafiltration units (Amicon Ultrafree MC-0.5 mL, Centrifugal Filter Unit 30 K, Merck Millipore, Milan, Italy) according to Villa et al. (1997). Spiked blood samples with cefazolin concentrations from 5 to 100 μg/mL were incubated for 30 min at 37°C before ultrafiltration. Subsequently, the sera were centrifuged (5000 *g*, 20 min) with 30000 Nominal Molecular Weight Limit (NMWL) cut-off ultrafiltration units and injected into a HPLC system. The binding percentage was calculated by serum (unbinding drug) and water (total cefazolin) peak area ratio.

### Population Pharmacokinetics and Monte Carlo Simulation

Pharmacokinetic modeling was carried out using commercially available software (Phoenix NLME version 7.0, Certara, St. Louis, MO, United States). A nonlinear mixed effects (NLME) approach was used to generate Pop PK parameter estimates. Two- and three-compartment models were evaluated to identify the model that best described the dataset. After visual inspection of plots for all dogs showing a polyphasic decay of plasma concentration vs. time, data were fitted with a two or three-compartment models. The two concurrent models were then compared using the likelihood ratio test (LRT) that is appropriate when models are nested (one model is a subset of another) and have different numbers of parameters. The critical value of the χ2 distribution to consider was obtained using Excel (Microsoft Office 2016) to estimate the risk of type one. Finally, a two-compartmental model was selected. Parameterization was in terms of serum clearance (CL), intercompartmental CL(s) (Q) and volume(s) of distribution (V) with V1, V2, CL, and Q being the primary estimated parameters. The following parameters were computed as secondary parameters.

The terminal slope *Beta* was obtained with Eq. 1;

$$Beta=0.5\times \left[\frac{Q}{V1}+\frac{Q}{V2}+\frac{CL}{V1}-{\left[{\left(\frac{Q}{V1}+\frac{Q}{V2}+\frac{CL}{V1}\right)}^{2}-4\frac{Q}{V2}\times \frac{CL}{V1}\right]}^{0.5}\right]$$

The initial slope of distribution, *Alpha*, was obtained with Eq. 2; $Alpha=\frac{Q}{V2}\times \frac{CL}{V1}/Beta$ and the elimination (HL_Beta_) and distribution (HL_Alpha_) half-lives were obtained with classical equations. The area under the curve (AUC), the steady-state volume of distribution (V_ss_) and the volume of distribution associated with the terminal phase (V_z_) were also computed as secondary parameters with classical equations.

The between-subject variability (BSV) was modeled using an exponential model (Eq. 3), and hence the CL for the *i*th subject was written as:

$$Cl_{i}=\theta_{\mathrm{median}}\times Exp(\eta_{i})$$

where *Cl*_i_ is the CL in the *i*th animal, θ_median_ is the population median CL (typical value of CL) and η_i_ the deviation (noted ETA) associated with the *i^th^* animal from the corresponding θ_median_ population value. Others individual parameters (i.e., V1, V2, and Q) were modeled using equations of the same form. The distribution of the ETAs was assumed to be normal with a mean of 0 and a variance (ω^2^_x_). In addition, the individual parameters and consequently their corresponding ETAs can be correlated. All these correlations were estimated and the corresponding covariances were stored in the variance-covariance omega (Ω) matrix. The following equation 4 was used to convert the variance (ω^2^_clearance_) of the log-transformed CLs into a coefficient of variation (CV %) in the original scale:

$$CV_{\mathrm{clearance}}(\%)=100\times \sqrt{\exp(\omega_{\mathrm{clearance}}^{2})-1}$$

The shrinkage of random effects toward the means was calculated for the ETAs (Karlsson and Savic, 2007) with equation 5:

$$Shrinkage=1-\frac{SD(EBE_{\eta })}{\omega }$$

where ω is the estimated variability for the population and SD is the standard deviation (SD) of the individual values of the empirical Bayesian estimates (EBE) of η.

The residual model was an additive plus a multiplicative (proportional) model of the form (Eq. 6):

$$C(t)=f(\theta ,\;Time)\times (1+\varepsilon_{1})+\varepsilon_{2}$$

with ε1 and ε2, the multiplicative and additive error terms having a mean of 0 and a variance noted σ1 or σ2, respectively. In Phoenix, when this error model is used, the additive sigma is reported as its SD, noted stdev, with the same units as serum concentration (μg/mL) and the multiplicative sigma is called multStdev and the 100^∗^ multStdev is the corresponding coefficient of variation.

Parameter estimation was based on minimizing an objective function value (OFV), using maximum likelihood estimation (i.e., minus twice the log of the likelihood) given for each model. The first order conditional estimation extended least squares (FOCE ELS) engine was used for analyses approximating the marginal likelihood while searching for the maximum likelihood. There was no censored data. A bootstrap approach was used to estimate typical values of parameters and precision of estimates that are reported as SE, CV % and by their 95% confidence intervals. To evaluate the overall performance of the final model, a visual predictive check (VPC) was plotted to compare actual observations with simulated replicates from the model (500 replicates per investigated dogs). The 90% prediction intervals were constructed and plotted together with the observed data allowing for a visual assessment of the agreement between simulation and observation. Diagnostic plots, the distribution of errors, and the precision of the parameter estimates were used as tools to evaluate the goodness of fit and to compare models.

The LRT was used to examine different models for testing the residual variability and the covariate effect on each PK parameter. An analysis of each covariate in all PK parameter was carried out to evaluate the significance on the model. The categorical covariates considered were the health status with two levels (healthy, diseased), the sex with three levels (male, female, and female neutered) and the breed with two levels (mongrel and other breeds) (Eq. 7):

$$Param=\theta_{\mathrm{median}}\times (1+\theta_{1}X_{1})$$

where *Param* is one of the structural parameter of the disposition model (V1, V2, CL, Q), *X*_1_ is an indicator variable with a value of 0 for control condition (the healthy condition for the health status, mongrel dog for breed), and of 1 for the non-mongrel breed and disease status. For example for V1, the model was given either by Eq. 8 for the healthy condition, or Eq. 9 for the diseased condition:

$$V1=\theta_{V1\mathrm{median}}\times EXP(\eta V1)$$

$$V1=\theta_{V1\mathrm{median}}\times (1+\theta_{1})\times EXP(\eta V1)$$

where θ_v1median_ is the typical value of V1, ηV1 is the ETAs associated with V1 and θ_1_,the fixed effect of the covariate for the diseased condition. If θ_1_ is significantly different from 0, it provides evidence that a difference exists between the healthy status and the disease condition for V1.

Age, BW, creatinine level and surgery time were considered as continuous covariates and their influence were modeled using a classical regression equation with a power model and an appropriate scaling factor for each covariate; for example serum CL was modeled with the following general equation 10:

$$CL=\theta_{1}\times {\left[\frac{Age}{8}\right]}^{\theta_{2}}\times {\left[\frac{BW}{20}\right]}^{\theta_{3}}\times {\left[\frac{CREAT}{0.9}\right]}^{\theta_{4}}\times {\left[\frac{Surgery\_time}{80}\right]}^{\theta_{4}}$$

where θ_1_ is the typical value of CL for a 8 years old dog, weighting 20 kg, having a creatinine level of 0.9 mg/dL and for a surgery time of 80 min. The stepwise covariate search mode was used to define the statistically significant covariates for each of the structural parameters of the model. This run mode performs a stepwise forward or backward addition or deletion of covariates effects (by adding one at a time) to determine the improvement of the final model based on the Bayesian information criterion (BIC). For the present analysis, we selected a BIC value of 6.635 for adding a covariate and a value of 10.823 for deleting a covariate, as these values are equivalent of *P* < 0.01 and *P* < 0.001 for the minus twice the log-likelihood (2-LL) criterion when using the LRT test (see **Supplementary File S2** for further details).

Using the previously developed Pop PK model and estimated parameters, 2500 curves of the cefazolin disposition were generated by the Monte Carlo simulation. The simulated dosage regimen was of 25 mg/kg IV at 6 h intervals over 24 h (i.e., four administrations of cefazolin at 0, 360, 720, and 1080 min). The 2500 curves were analyzed with the non-compartmental tool of Phoenix. The duration for which free plasma concentrations were above the selected MICs (from 0.25 to 8 mg/L) was computed using the statistical tool of Phoenix. The quantiles 90 and 95% of the distributions of these different times above MIC were computed to give the corresponding probability of target attainment (PTA) of the selected index (*f*T > MIC for 50% of the dosing interval). The PTAs were computed without their confidence intervals.

## Results

### Animals and Cefazolin Concentrations

Seventy-eight dogs were enrolled in the study; animal characteristics are reported in **Table 1** together with the covariates and coding used for Pop PK modeling. Most dogs had some conditions and were undergoing different types of surgery from oncologic to ophthalmic; only 19 dogs were healthy and undergoing gynecological or andrological surgery. The dogs of the study represented many different breeds (*n* = 26), from Jack Russell Terriers to Pyrenean Sheepdog; among these, 12 breeds were represented by more than 1 dog and 14 were represented by only one dog.

**Table 1:** Animal characteristics, covariates and coding used in Pop PK analysis.

	Mean ± S.D.	Range (median)	
**Continuous covariates**			
Age (y)	7.22 ± 4.11	0.66–14 (8)	
Body weight (kg)	26.13 ± 0.88	4.5–56 (27)	
Creatinine level (mg/dL)	0.91 ± 0.32	0.3–1.88 (0.9)	
Surgery Time (min)	87.63 ± 58.09	20–260 (80)	

	**Type and number of subjects (Code)**		

**Categorical covariates**			
Health status	Healthy *n* = 19 (Code 0)	Diseased *n* = 59 (Code 1)	
Breed	Mongrel *n* = 27 (Code 0)	Other breeds *n* = 51 (Code 1)	
Sex	Male *n* = 32 (Code 0)	Female *n* = 23 (Code 1)	Female neutered *n* = 23 (Code 2)


**FIGURE 1:**
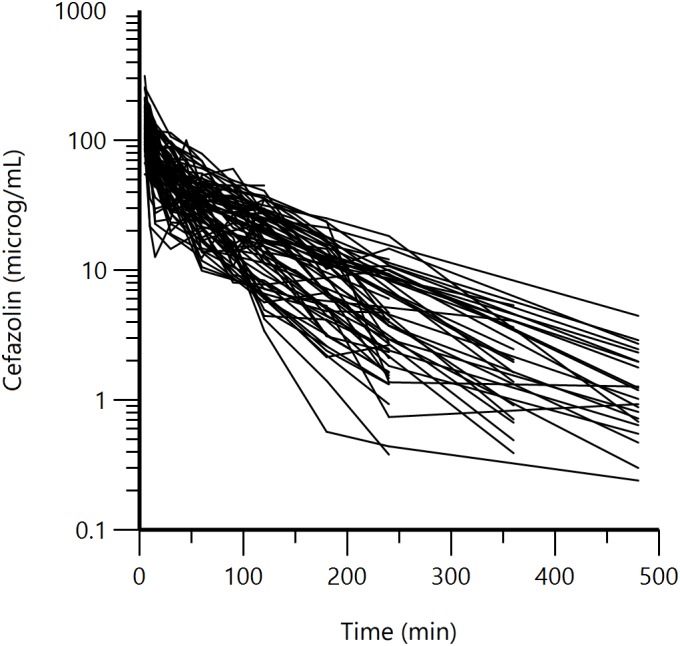
Semi-logarithmic spaghetti plots of the disposition curves of cefazolin over 480 min after a single IV bolus administration (25 mg/kg) in 78 dogs.

Two to 11 (median 9) blood samples were collected from each dog from 5 to 480 min for 14 different sampling times for a total of 629 collected samples. Cefazolin serum concentrations obtained in all animals are reported in **Figure 1** and a biexponential decay over time is shown. The percentage of cefazolin binding to serum protein, calculated with the ultrafiltration method, resulted in 36.2 ± 5.3%.

### Population Pharmacokinetics and Monte Carlo Simulation

**FIGURE 2:**
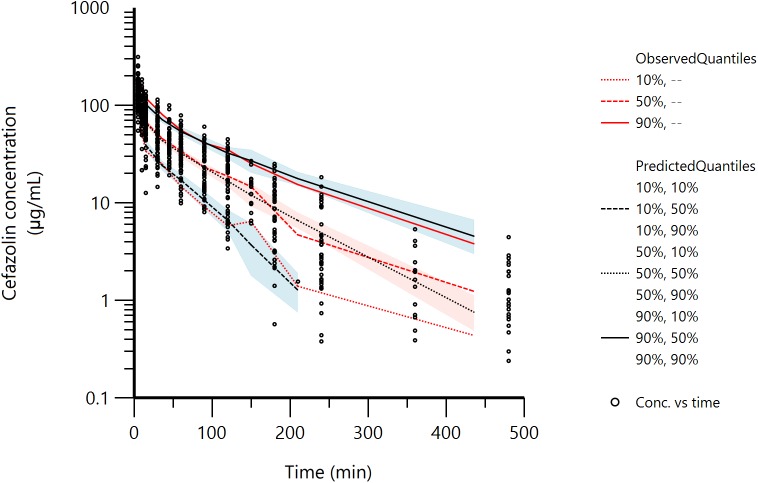
Visual Predictive Check (VPC) plot obtained with 500 replicates of each animal (314500 simulated data). Red lines: observed quantiles; Black lines: predicted quantiles; Black circles: observed data. The shaded areas represent the 90% confidence intervals around the 10th, 50th, and 90th percentiles of the simulated data.

**FIGURE 3:**
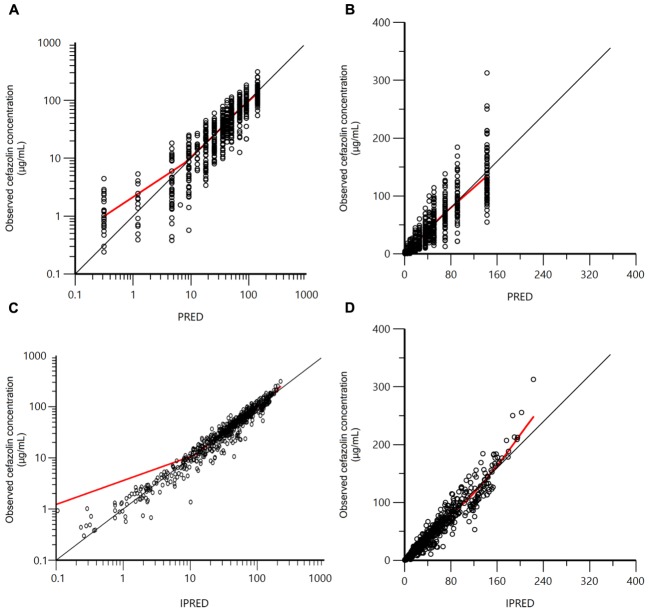
Plots of the observed cefazolin concentration (μg/mL) versus population predicted concentration (PRED; μg/mL – **A** in logarithmic scale; **B** in arithmetic scale) and versus individual predicted concentration (IPRED; μg/mL – **C** in logarithmic scale; **D** in arithmetic scale).

The two-compartment model was adequate to describe cefazolin disposition in our dogs, as shown in the VPC plot (**Figure 2**) and in the plots of the observed cefazolin concentration versus population predicted concentration (PRED) or versus individual predicted concentration (IPRED) (**Figures 3A–D**). The model adequacy was further supported by the inspection of different goodness-of-fit plots (see **Supplementary Figures S1—S4**).

Typical values of the primary structural parameters of the model, secondary parameters, their associated standard error (SE) and the SD of the residual for the basic model are given in **Table 2**.

**Table 2:** Population primary and secondary parameters of cefazolin in dogs obtained with a two-compartment model (bootstrap estimates of mean, median, SE, CV%, 2.5%, and 97.5% percentiles).

Parameters	Units	Mean	SE	CV%	Median	2.5%	97.5%
**Population primary parameters**							
tvV1	L/kg	0.116	0.013	11.36	0.115	0.084	0.137
tvV2	L/kg	0.177	0.011	6.01	0.176	0.158	0.194
tvCL	L/kg/min	0.0037	0.0002	4.26	0.0036	0.0034	0.0040
tvQ	L/kg/min	0.0103	0.0013	12.82	0.0105	0.0073	0.0123
tvCMultStdev		0.257	0.016	6.08	0.256	0.226	0.285
stdev (sigma)	μg/mL	0.564	0.166	29.42	0.543	0.314	0.943
**Secondary parameters**							
AUC	μg^∗^min/mL	6790	286	4.21	6810	6189	7391
Beta	1/min	0.0111	0.0006	5.29	0.0111	0.0100	0.0122
Beta half-life	min	57.93	3.11	5.38	57.72	52.45	63.84
Alpha	1/min	0.172	0.031	18.21	0.171	0.115	0.241
Alpha half-life	min	4.17	0.77	18.45	4.06	2.88	6.04
Vss	L/kg	0.292	0.013	4.33	0.293	0.266	0.316
MRT	min	79.34	4.25	5.36	79.25	71.74	86.12
Vz	L/kg	0.334	0.016	4.71	0.335	0.305	0.365


*Typical values (tv); V1, volume of the central compartment; V2, volume of the peripheral compartment; CL, serum CL; Q, intercompartmental CL; CmultStdev, multiplicative component of the residual that can be read as a coefficient of variation of 25.7%; stdev, standard deviation of the additive component of the residual; AUC, area under the curve; Beta, slope of the terminal phase; Beta half-life, half-life of elimination; Alpha, slope of the distribution phase; Alpha half-life, half-life of distribution; MRT, mean residence time; Vz, volume of distribution associated with the terminal phase; SE, standard error of estimate.*

For BSV, an exponential model was selected, because the estimated thetas parameters must be positive and their distribution is generally right-skewed. The estimates of the random effects variance-covariance matrix, correlation matrix and shrinkage are reported in **Table 3**. The full variance-covariance omega (Ω) matrix was selected, because in a mixed effect model, inclusion of covariance terms prevented the risk of biased estimation of the variance terms (the diagonal). It also allowed for checking possible correlations between the ETAs that would suggest some over parameterization of the model (statistical collinearity). Inspection of **Table 3** showed no spurious correlation between ETA and low values for ETA shrinkage indicating that data were rich enough to properly estimate the random component of the model.

**Table 3:** Estimates of the random effects variance-covariance matrix, correlation matrix and shrinkage.

**Omega**				
nV1	**0.092598**			
nV2	0.099641	**0.135063**		
nCL	0.031062	0.073881	**0.130511**	
nQ	0.00518	0.062008	0.068201	**0.196614**
**Correlations between ETAs and Shrinkage**				
nV1	**1**			
nV2	0.89	**1**		
nCL	0.28	0.56	**1**	
nQ	0.04	0.38	0.43	**1**
Shrinkage	0.15	0.11	0.05	0.34


*Variance (diagonal) is in bold. nV1, nV2, nCL and nQ are the random component of the model (ETA). Corresponding BSV expressed as a CV% are 31.15, 38.03, 37.34, and 46.61% for nV1, nV2, nCl, and nQ, respectively (see equation 4).*

The bootstrap estimate of the BSV for the 4 primary parameters and precision of their estimate (expressed as CV%) were also calculated and are reported in **Table 4**. Inspection of **Table 4** indicates that bootstrap estimates of the BSV were consistent with those obtained by a single run of all the data set (**Table 3**) and that the precision of the BSV estimates were robust (low CV%). The BSV for CL (the parameter controlling the overall cefazolin exposure) was rather high (CV = 36.83%) and prompted us to explore the influence of different clinically relevant covariates to explain the variability in the observed, large clinical population.

**Table 4:** The bootstrap estimates of the Between Subject Variability (BSV) for the 4 primary parameters and precision of their estimate (expressed as CV%).

	BSV (%)	Precision (CV%)
nV1	31.42	24.00
nV2	42.70	22.34
nCL	36.83	12.80
nQ	46.12	26.48


*nV1, nV2, nCL, and nQ are the random component of the model (ETA). Corresponding BSV is expressed as a CV%, as well as precision of the estimate.*

The significant influence of each covariate (sex, age, breed, BW, health status, creatinine level, and surgery time) on the model was explored with a stepwise covariate search mode. The diseased condition was defined based on clinical exam, anamnesis and hematological and biochemical blood tests. As cefazolin is mainly excreted by the kidney (approximately 80%, Nishida et al., 1970) the creatinine levels was used as a covariate to assess individual kidney conditions. Continuous covariates were scaled to avoid instability of the optimization process and to provide parameter estimates that were more reflective of the average subject; the scaling values were BW = 20 kg, age = 8 years, creatinine level = 0.9 mg/dL and surgery time = 80 min. The scatter plot matrix for the continuous covariates (age, BW, creatinine level, and surgery time) is reported in **Supplementary Figure S5**. The visual inspection of the figure shows no obvious relationship, but there was a trend between creatinine level and age and BW. The scatter plot matrices for the continuous covariates (age, BW, creatinine level, and surgery time) *per* health status level (0 = healthy, 1 = disease) are reported in **Supplementary Figures S6A,B**. Moreover, in this case, the visual inspection of the figure shows no obvious relationship.

The Phoenix stepwise search exploratory tool returned 27 combinations (scenarios) of covariates, ensuring a statistically significant (*P* < 0.01) reduction of the BIC criterion. The most significant scenarios were those related to V1 and for 16 scenarios that included two covariates, the BW was selected as the covariate. Among these scenarios, the most relevant to perform subsequent simple runs were chosen and fitted to estimate the magnitude of the effect. Finally, to assess whether these statistically significant covariates had clinical relevance and merit for future recommendation or warning, the influence of covariates on PK parameters (V1, V2, CL, Q) was explored by computing the multiplicative/dividing factor when the covariate increased or decreased by 50%.

For example for BW as a covariate, we computed the values of PK parameters for a typical dog of 20 kg BW (see equation 10). When only the BW influenced the CL, the typical value of the fixed effect (θ_2_in equation 10) was of -0.2368. This means that for dogs of 10 and 30 kg BW, i.e., for dogs having a BW of plus or minus 50% of the scaled typical value, the typical value of CL was increased of time folds a factor of 1.178 for a dog of 10 kg BW and was multiplied by 0.908 in a dog of 30 kg BW (or equivalently divided by 1.10). Such a difference can be considered as not relevant from a clinical point of view (see **Supplementary File S2** for further details).

For age, BW, creatinine level and surgery time, the influence was not clinically relevant. For the health status, all PK parameters were influenced, but the largest effect was observed for the Q (-0.267), meaning that in diseased dogs, the intercompartmental CL that likely reflects tissular blood flow, was decreased by 26.7% compared with that in control dogs. Other influences of disease were clinically irrelevant. For breed and sex, the magnitude of the effects was also clinically irrelevant despite their statistical significance. It has to be said that, statistical significance does not always mean clinical relevance, as especially in this trial where rich and robust data were analyzed, allowing to easily detect statistically significant differences but having no clinical impact.

By Monte Carlo simulation, 2500 curves were generated using this Pop PK model to compute the PTA corresponding to the selected possible MIC in order to propose a PK/PD cutoff, considering the average percentage of unbound drug calculated (i.e., 0.64; percentage of cefazolin binding 36 ± 0.53%). **Table 5** reports the results of *f*T > MIC for the 2500 curves simulated for a dosing regimen of 25 mg/kg at 6 h interval over 24 h. In **Table 5** it is shown that for a MIC of 2 mg/L (i.e., a total serum concentration of 3.12 mg/L, corrected for the unbound fraction of the drug), 90% of dogs had a *f*T > MIC of 57% over the dosage interval (24 h). Thus, the PK/PD cutoff for a *f*T > MIC target of 50% of the dosage interval and a 90% quantile (or 95%) is set at 2 mg/L.

**Table 5:** Time (min) above possible MICs ranging from 0.25 to 8 mg/L corresponding to total serum concentration ranging from 0.39 to 12.5 mg/L for the quantiles (Q) 90 and 95% and corresponding value of the T > MIC in % of 24 h.

MIC (mg/L)	Total serum concentration (MIC/unbound fraction)	Time min (Q90%)	T > MIC (%)	Time min (Q95%)	T > MIC (%)
0.25	0.39	1345	93.4	1197	83.1
0.5	0.78	1169	81.2	1062	73.7
1	1.56	882	61.2	787	54.6
**2**	**3.12**	**821**	**57.0**	**735**	**51.1**
4	6.25	530	36.8	468	32.5
8	12.5	463	32.1	407	28.3


*Q90%: quantile 90%; Q95%: quantile 95%; for a MIC of 2 mg/L corresponding to a total serum concentration of 3.12 mg/L, 90% of the simulated curves were equal or above a free plasma concentration for 821 min, i.e., for 57% of the considered dosing interval.*

## Discussion

Cefazolin is very commonly used perioperatively and has been recommended as an appropriate prophylactic antimicrobial for dogs undergoing surgery. Currently, its use is based on not recent studies with classical PK investigation that do not consider the possible large inter-animal variability encountered in clinical practice nor the most recent PK/PD paradigms allowing to support the prudent use of the AMD at a population level. Thus, by developing a Pop PK model, the aim of this study was to estimate typical PK parameters of cefazolin, their BSVs and to identify whether covariates such as sex, age, BW, breed, health status, creatinine level, and surgery time, have an influence on these parameters and in turn, to explain the BSV of cefazolin disposition. Moreover, to promote a responsible use of cefazolin in dogs, the study aimed to compute a PK/PD cutoff value for the subsequent determination of a specific CBP for the development of an AST, using the VetCAST approach (Toutain et al., 2017).

A large number of dogs were enrolled in the study (*n* = 78) with variable characteristics reflecting the target clinical population of dogs undergoing surgery. Only 19 dogs were healthy, and among the diseased dogs, 60% were oncologic patients. Many different breeds were represented in the study together with a large number of mongrels, accounting for the wide variability encountered in clinical practice. Many blood samples were taken from each animal; thus, the large availability of data (*n* = 629 samples) made its analysis reliable, especially for the estimation of the BSV that require a minimal number of samples per animal to avoid an ETA-shrinkage, i.e., the individual parameter estimates “shrink” back toward the population parameter estimate (Karlsson and Savic, 2007). In the present trial, all ETA-shrinkages were rather low giving confidence in the value of the individual EBE (individual ETA) and *post hoc* computations.

After cefazolin administration, a biexponential decay was observed in our samples, as also reported by other authors (Rosin et al., 1993; Marcellin-Little et al., 1996; Singh et al., 1998; Harika et al., 2001), and the two-compartment model was the most adequate to describe cefazolin disposition in dogs. The percentage of cefazolin binding to protein was 36.2 ± 5.3%. This value was in agreement with a previous study reporting 38.8 ± 2.51% from a bioassay and 35.8 ± 2.64% from isotopic methods (Daly et al., 1982).

The primary and secondary Pop PK estimates were in agreement with the results obtained with classical PK modeling reported by other authors, although all were obtained with different analytical techniques (microbiological assay vs. HPLC) or with different doses (Rosin et al., 1993; Marcellin-Little et al., 1996; Singh et al., 1998; Harika et al., 2001). For example, elimination half-life was (typical value ± SE) 57.93 ± 3.11 min in our study vs. 55.08 ± 7.92 min (mean ± SD) in the study by Rosin et al. (1993) or 52.3 min by Marcellin-Little et al. (1996) when administered via IV at 20 or 22 mg/kg, respectively. In contrast, only a population investigation allows for proper estimation of a BSV reflecting altogether the main sources of variability encountered in all-coming dogs. For example, the BSV as it can be roughly estimated from the mean and SD reported by Rosin et al. (1993) is of approximately 14.4%, while from our 78 dogs, considering the *post hoc* estimates of individuals ETAs and solving equation 1 to compute the terminal half-life, its BSV was estimated at 31.2%. This point should be highlighted when computing the PK/PD cut off to establish a CBP for an AST using the VetCAST approach, because AST should *a priori* cover most individuals within the targeted population, not only a limited number of experimental dogs. We were also in position to investigate the influence of the different measured covariates. This is of relevance when establishing a CBP, because the identification of a subpopulation could lead to some specific comments to assist clinical microbiologists in the routine interpretation of AST data and in suggesting the most appropriate actions to be taken in response to AST results. In addition, identification of a subpopulation could lead to some specific recommendation in terms of dosing regimen.

The influences of age, BW, creatinine level and surgery time on the exposure of cefazolin were not clinically relevant. Health status statistically influenced all primary PK parameters, with the most evident effect being on the decrease in the intercompartmental CL in diseased dogs; nevertheless, this influence can be considered too low a magnitude to be clinically relevant. Overall, the Pop PK analysis performed showed that the 25 mg/kg dosage provides consistent cefazolin exposure in a wide range of canine patients and no adjustment of dose for special dog populations seems necessary.

Dosage recommendations for surgical prophylaxis administration of cefazolin to dogs varied from 20 to 25 mg/kg IV at beginning of surgery or 30 min before, followed by 20 mg/kg IV every 60 or 90 min until wound closure or 20 mg/kg SC at 6 h (Rosin et al., 1993; Whittem et al., 1999; Plumb, 2011). The practice of frequent repetition of cefazolin administration during surgery has been suggested to produce very high serum concentrations (10 × MIC; i.e., 20 mg/L) to prevent infection from skin contaminants (Marcellin-Little et al., 1996). Nevertheless, for a time-dependent AMD, like cefazolin, these very high concentrations may be unnecessary, and a standard PK/PD target is to maintain plasma concentration above the MIC_90_ of putative pathogens for a least 50% of a dosing interval (Turnidge, 1998; Toutain et al., 2002; Papich, 2014). The bacteria most commonly involved in SSIs of dogs are commensal organisms on the skin such as *Staphylococcus pseudintermedius*, a gram-positive bacterium for which a CBP of 2 mg/L has been proposed by the Clinical Laboratory Standards Institute (CLSI) for skin and soft tissue infections with a dosage regimen of 25 mg/kg administered every 6 h (Clinical Laboratory Standards Institute [CLSI], 2015). The present trial is consistent with such a CBP. By using our population model and Monte Carlo simulations, we established a PK/PD cut off of 2 mg/L for a dosage of 25 mg/kg administered every 6 h (4 administration in 24 h) and a target *f*T > MIC set at 50% of the dosing interval to be achieved in at least 90% of a representative dog population.

## Conclusion

In conclusion, from a PK/PD perspective, the present population investigation supports cefazolin use for empirical prophylactic administration to dogs 30 min before surgery with possible readministration at 6 h interval for pathogens with a MIC ≤ 2 mg/L.

## Ethics Statement

This study was carried out in accordance with the European Directive (2010/63/UE) and Italian law (D.L. 26/2014). The protocol was approved by the ORGANISMO PREPOSTO AL BENESSERE DEGLI ANIMALI (OPBA), Università degli Studi di Milano.

## Author Contributions

PC, FDC, and RV designed the trial. FDC and GR conducted the experimental phase. FDC and PC conducted HPLC analysis. P-LT and PC conducted Pop PK analysis. P-LT and AB-M conducted the Monte Carlo simulation and calculated the PK/PD cut off. FDC, PC, and P-LT drafted the paper. All the co-authors critically reviewed several drafts and approved the final manuscript.

## Conflict of Interest Statement

The authors declare that the research was conducted in the absence of any commercial or financial relationships that could be construed as a potential conflict of interest.

## Supplementary Material

FIGURE S1 Plot of conditional weighted residuals (CWRES) against time.

FIGURE S2 Plot of conditional weighted residuals (CWRES), against population prediction of cefazolin concentrations (PRED).

FIGURE S3 Plot of weighted residuals (CWRES) against time.

FIGURE S4 Plot of weighted residuals (CWRES), against population prediction of cefazolin concentrations (PRED).

FIGURE S5 Scatter plot matrix for the continuous covariates, age, BW, creatinine level and surgery time.

FIGURE S6 **(A,B)** Scatter plot matrices for the continuous covariates (age, BW, creatinine level and surgery time) per health status level (0 = healthy, 1 = disease).

FILE S1 Description of the cefazolin analytical method.

FILE S2 Additional details on Population Pharmacokinetic analysis.
