# Supplementary material for: Population Pharmacokinetic Study of Cefazolin Used Prophylactically in Canine Surgery for Susceptibility Testing Breakpoint Determination
Source: Front Pharmacol. 2018 Oct 9;9:1137. doi: 10.3389/fphar.2018.01137 (PMC6190795; doi:10.3389/fphar.2018.01137)
Supplement: FIGURE S1 — Plot of conditional weighted residuals (CWRES) against time. [file Table_3.docx]

Supplementary Material

**Population pharmacokinetic study of cefazolin used prophylactically in canine surgery for susceptibility testing breakpoint determination**

Petra Cagnardi*, Federica Di Cesare, Pierre-Louis Toutain, Alain Bousquet-Mélou, Giuliano Ravasio, Roberto Villa

*** Correspondence: Petra Cagnardi: petra.cagnardi@unimi.it**

# Supplementary Figures S1-S6


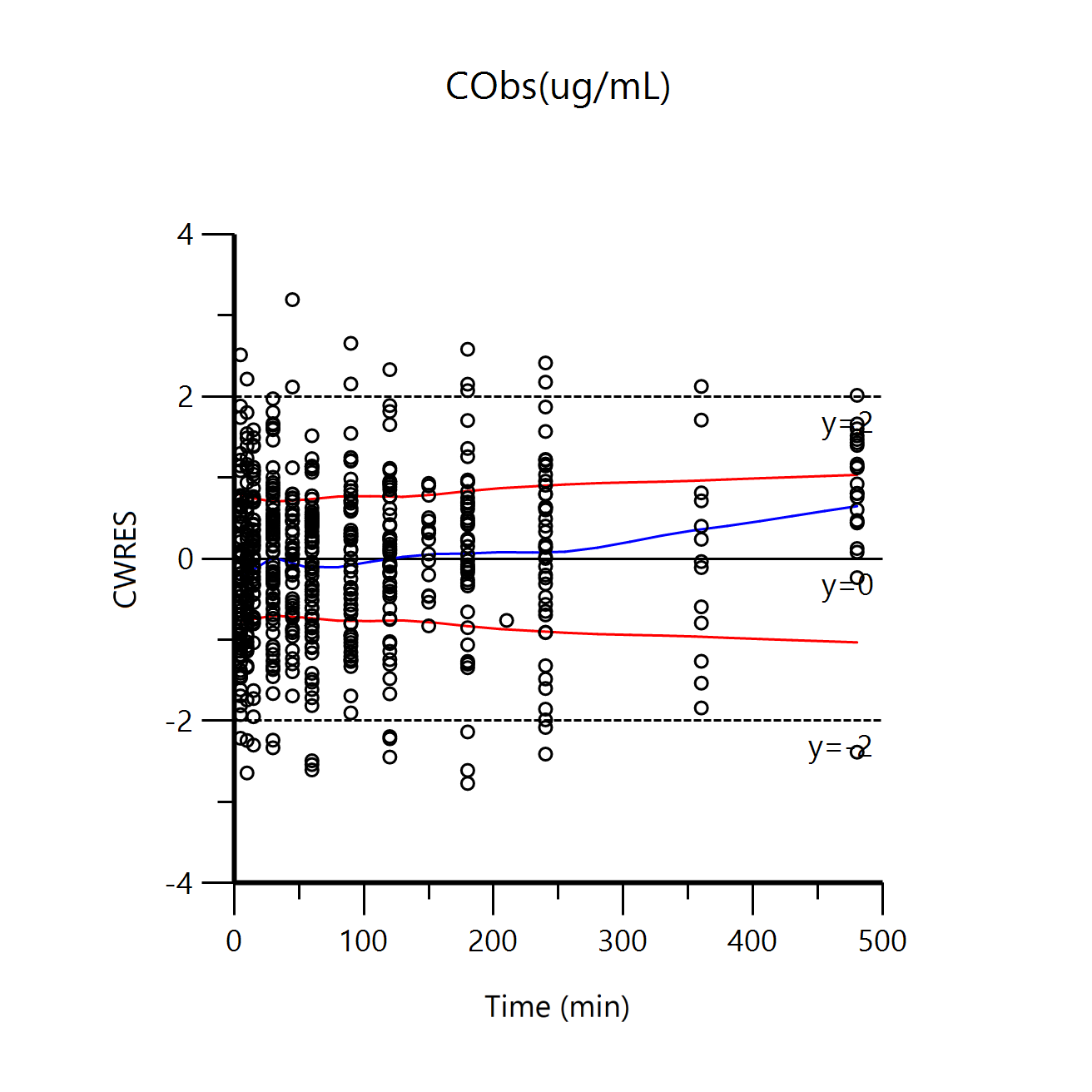


**Figure S1:** Plot of conditional weighted residuals (CWRES) against time.

**
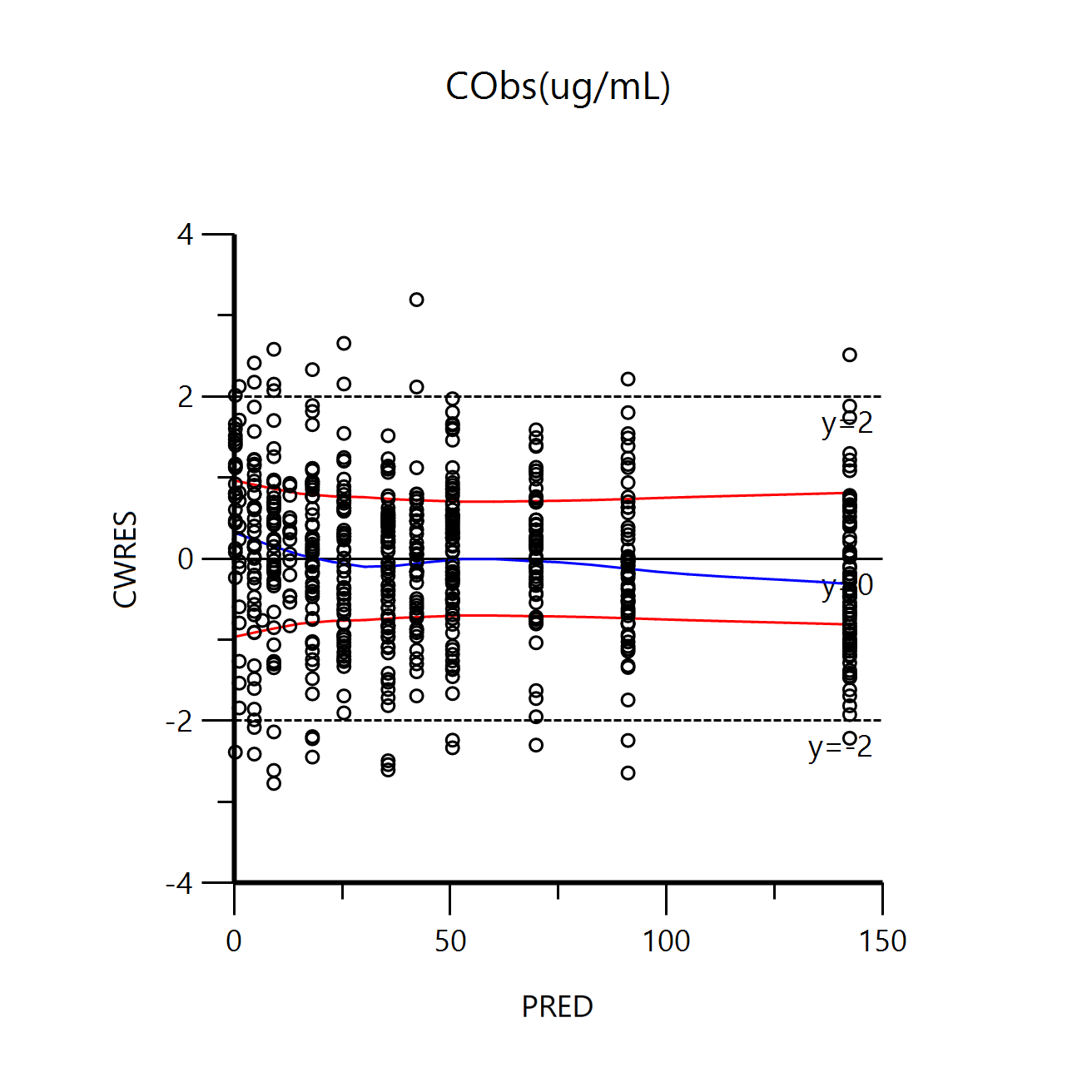
**

**Figure S2**. Plot of conditional weighted residuals (CWRES), against population prediction of cefazolin concentrations (PRED).


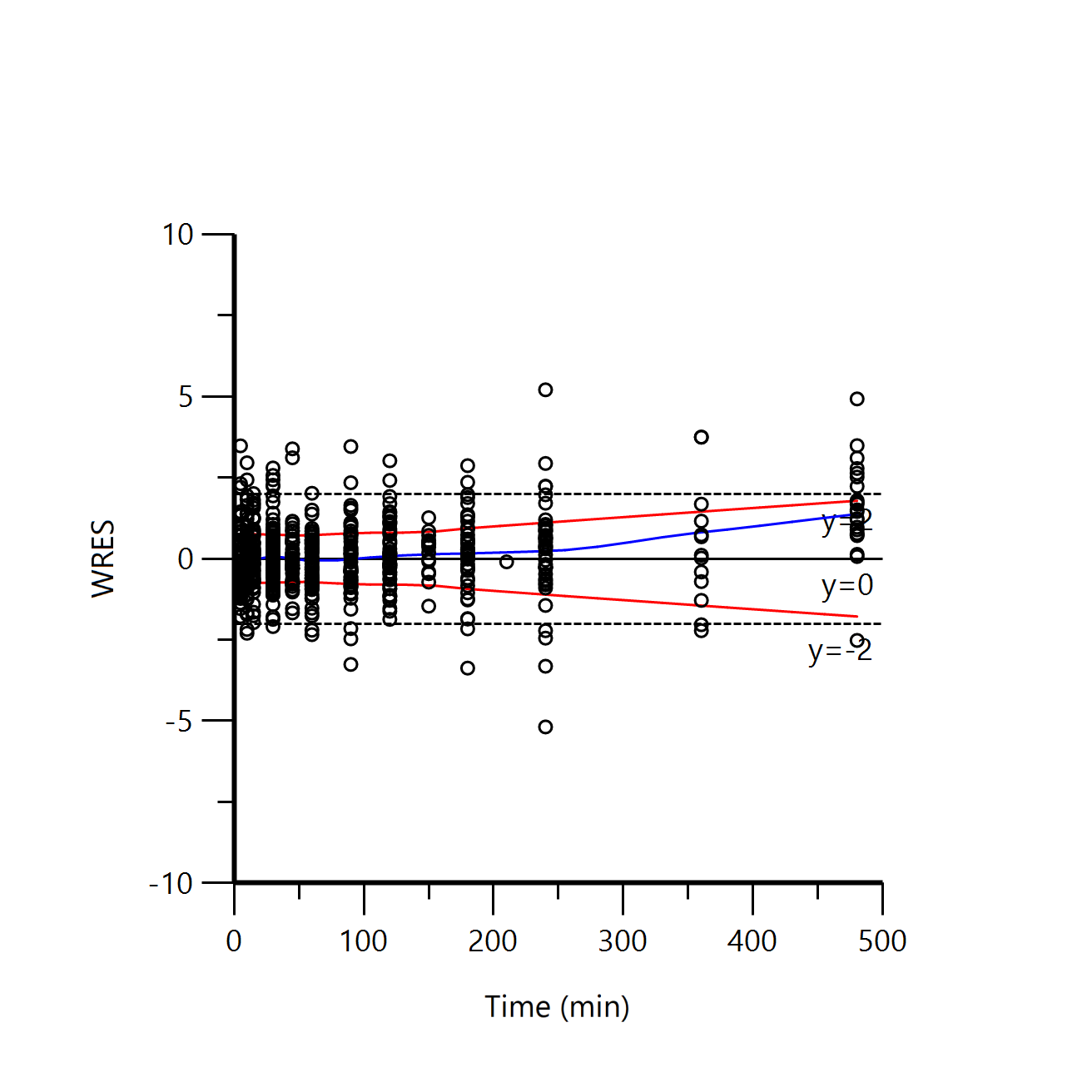


**Figure S3**: Plot of weighted residuals (WRES) against time.


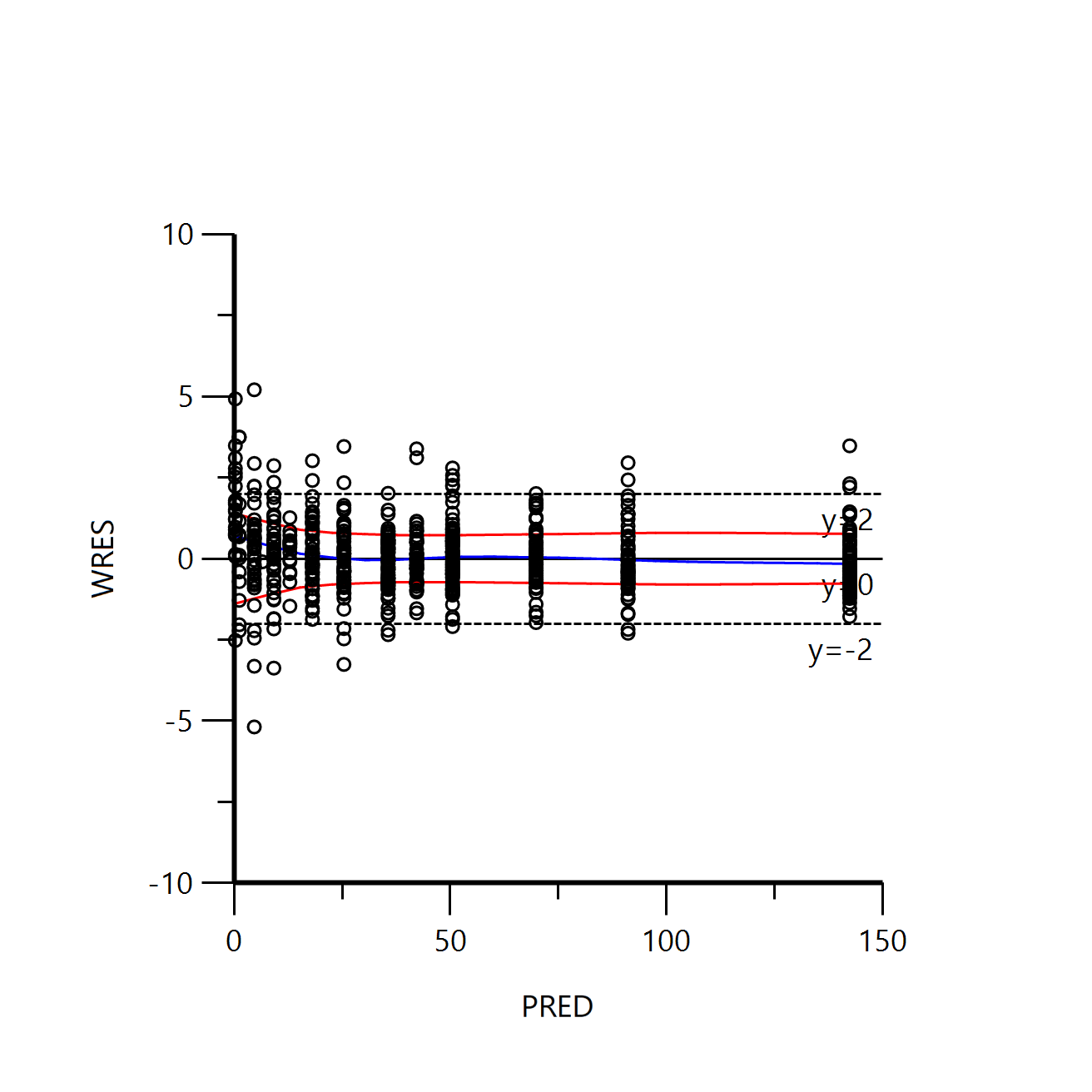


**Figure S4**: Plot of weighted residuals (CWRES), against population prediction of cefazolin concentrations (PRED).

**Figure S5:** Scatter plot matrix for the continuous covariates, age, BW, creatinine level and surgery time.

**A**

**B**

**Figure S6 A and B** the scatter plot matrices for the continuous covariates (age, BW, creatinine level and surgery time) per health status level (0 = healthy, 1 = disease).

**
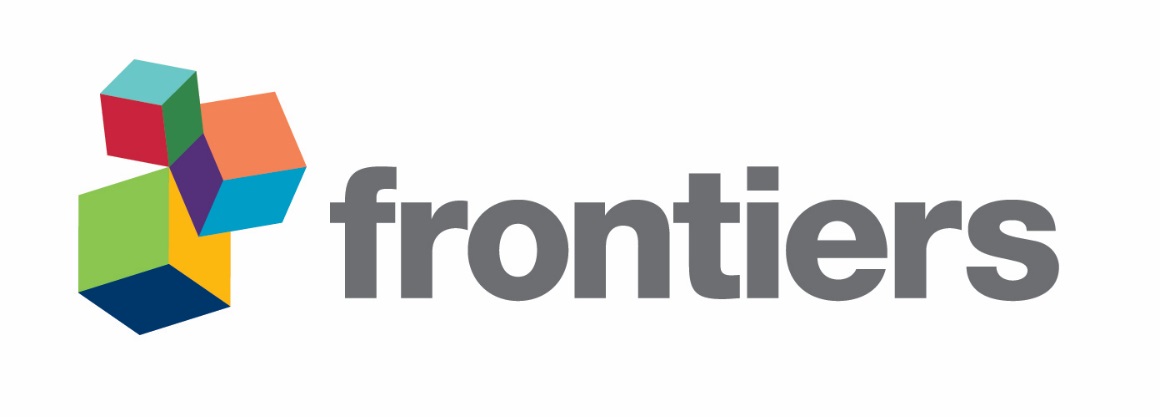
**
