# Supplementary material for: Population Pharmacokinetic Study of Cefazolin Used Prophylactically in Canine Surgery for Susceptibility Testing Breakpoint Determination
Source: Front Pharmacol. 2018 Oct 9;9:1137. doi: 10.3389/fphar.2018.01137 (PMC6190795; doi:10.3389/fphar.2018.01137)
Supplement: FILE S1 — Description of the cefazolin analytical method. [file Table_1.DOCX]

Supplementary Material

**Population pharmacokinetic study of cefazolin used prophylactically in canine surgery for susceptibility testing breakpoint determination**

Petra Cagnardi*, Federica Di Cesare, Pierre-Louis Toutain, Alain Bousquet-Mélou, Giuliano Ravasio, Roberto Villa

*** Correspondence: Petra Cagnardi: petra.cagnardi@unimi.it**

# Materials and Method

# Supplementary Material on cefazolin analytical method for extraction and quantification

Two hundred µL of canine serum were transferred to a 1.5 mL standard Eppendorf tube, then mixed with 400 µL of acetonitrile to precipitate proteins and the sample was vortexed for 30 s. After centrifugation (12,000 g, 10 min), the supernatant was transferred to a 10 mL Pyrex conical glass tube and evaporated to dryness by a centrifugal evaporator at 30° C. Then the dried extract was reconstituted in 100 µL of mobile phase and 20 µL were injected into the column. The cefazolin serum quantification was performed by an HPLC system that included a binary pump, an autosampler, a Peltier column oven set at 20°C and an UV/Visible detector (Series 200, Perkin Elmer, Milan, Italy) set at 272 nm of wavelength.

The drug separation was achieved by Accucore XL column C18 (250x4.6, 4 μm, Thermo Scientific, Milan, Italy) with adequate pre-column. The mobile phase consisted of a mixture of acetonitrile (A), water (B) and potassium dihydrogen phosphate KH_2_PO_4_ (0.5 M) (C) (A:B:C, 110:884:6, v/v) with a flow rate of 0.8 mL/min.

The analytical standard of cefazolin sodium salt (purity grade 96.8%) was provided by Sigma Aldrich (Milan, Italy). All reagents and solvents were purchased from VWR (Milan, Italy).

**
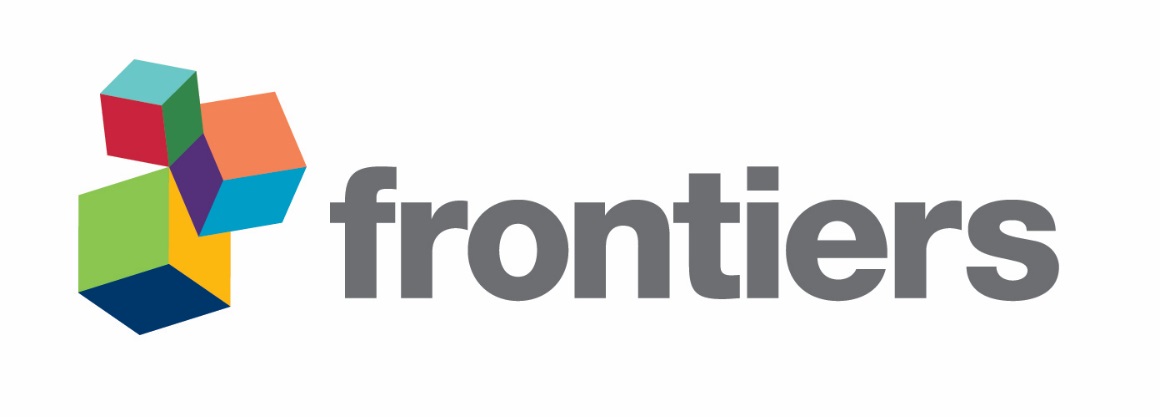
**
