# Supplementary material for: Population Pharmacokinetic Study of Cefazolin Used Prophylactically in Canine Surgery for Susceptibility Testing Breakpoint Determination
Source: Front Pharmacol. 2018 Oct 9;9:1137. doi: 10.3389/fphar.2018.01137 (PMC6190795; doi:10.3389/fphar.2018.01137)
Supplement: FILE S2 — Additional details on Population Pharmacokinetic analysis. [file Table_2.DOCX]

Supplementary Material S2

**Population pharmacokinetic study of cefazolin used prophylactically in canine surgery for susceptibility testing breakpoint determination**

Petra Cagnardi*, Federica Di Cesare, Pierre-Louis Toutain, Alain Bousquet-Mélou, Giuliano Ravasio, Roberto Villa

*** Correspondence: Petra Cagnardi: petra.cagnardi@unimi.it**

# Materials and Method

# Supplementary Material on Population Pharmacokinetic analysis

The Phoenix Cov. Srch. Stepwise (stepwise covariate search) run mode was used to search, without a priori, what are the statistically significant covariates for each of the structural parameter of the model. This run mode performs an automatic stepwise forward or backward addition or deletion of covariates effects by adding one at a time to determine if they make a sufficient threshold improvement based on the specified criterion options. The Bayesian Information Criterion (BIC) was the criterion chosen:

$$BIC=OBJ+n_{p}LN\left( N \right)$$

where np is the total number of parameters in the model, and N is the number of data observations. BIC penalizes the OBJ for model complexity more than the classical AIC, and may be preferable when data are limited. Kass and Raftery (Kass & Raftery. Bayes Factors, Journal of the American Statistical Association, 1995, 90:430, 773-795), categorized differences in BIC between models of >10 as “very strong” evidence in favor of the model with the lower BIC; 6 - 10 as “strong” evidence; 2 - 6 as “positive” evidence; and 0 - 2 as “weak” evidence. For the present analysis we selected a value of 6.635 for adding a covariate and a value of 10.823 for deleting a covariate. These two threshold values are equivalent of P<0.01 and P<0.001 for the minus twice the log-likelihood (2-LL) criterion when using the LRT. The structural model is used as a baseline and the covariate model is made increasingly complex. After each model estimation, the covariates are evaluated to see which one has the greatest improvement in the goodness-of-fit statistic selected greater than the BIC specified threshold. That covariate is added to the regression model for the structural parameter and the model is estimated. This process is repeated until all significant effects are accounted for. Then the process works in reverse to eliminate covariates on parameters whose removal produces the smallest reduction in goodness-of-fit less than the specified BIC threshold.

# Results

# Supplementary Material on Population Pharmacokinetic analysis

To assess the influence of a quantitative covariate (BW, age, creatinine and surgery time), we computed the multiplicative/dividing factor when the covariate is increase or decrease of 50 % respectively.

For example for BW, we computed the values of PK parameters influenced by BW having an estimated typical value (tv) of θ1, this tv being for a typical dog of 20 kg BW because it is our scaling BW value in the model. Thus, for dogs of 10 and 30 kg BW, i.e. for dogs having a BW of plus or minus 50% of the scaled 20 kg BW dog, the two next equations give for the volume of distribution the typical value for this parameter:

$$V1=tv\theta_{1}\times\left[ \frac{10}{20} \right]^{-0.21}=\theta_{1}\times1.156$$

$$V1=tv\theta_{1}\times\left[ \frac{30}{20} \right]^{-0.21}=\theta_{1}\times0.8645$$

For the scenario where only the BW influenced the **Clearance** (CL), the typical value of the fixed effect was of -0.2368 meaning that the tv of CL is increased of time folds a factor of 1.178 for a dog of 10 Kg BW and was multiplied by 0.908 in a dog of 30 Kg BW (or equivalently divided by 1.10). Such a difference can be considered as not relevant from a clinical point of view.

For three of the tested scenarios, **creatinine** actually influenced the plasma clearance value with a typical value of the exponent ranging from -0.18 to -0.23 meaning that creatinine, as BW, has a non-relevant influence on plasma clearance (multiplicative or dividing factor ranging from 1.13 to 1.17).

For **age** tv of the exponent ranged from -0.092 to -0.035 meaning multiplicative/dividing factor from 1.02 to 1.06 i.e. not relevant.

For the surgery time, exponents were of 0.0011 and 0.093 meaning multiplicative /dividing factor of 0.999 and 0.93 respectively without practical consequences.

**
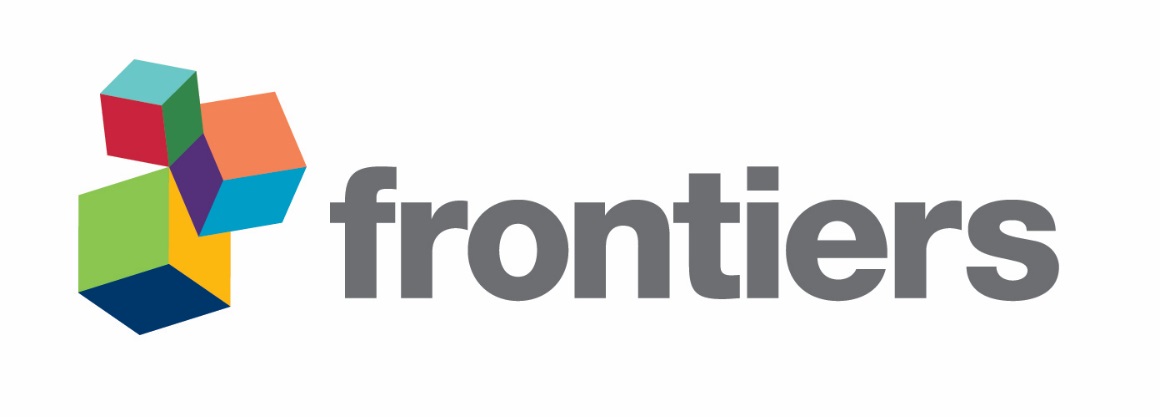
**
